# Arginine-directed glycation and decreased HDL plasma concentration and functionality

**DOI:** 10.1038/nutd.2014.31

**Published:** 2014-09-01

**Authors:** L Godfrey, N Yamada-Fowler, J Smith, P J Thornalley, N Rabbani

**Affiliations:** 1 Clinical Sciences Research Laboratories, Medical School, University of Warwick, University Hospital, Coventry, UK; 2 Bruker UK Ltd, Banner Lane, Coventry, UK

## Abstract

**Background/Objectives:**

Decreased plasma concentration of high-density lipoprotein cholesterol (HDL-C) is a risk factor linked to increased risk of cardiovascular disease (CVD). Decreased anti-atherogenic properties of HDL are also implicated in increased CVD risk. The cause is unknown but has been linked to impaired glucose tolerance. The aim of this study was to quantify the modification of HDL by methylglyoxal and related dicarbonyls in healthy people and patients with type 2 diabetes characterise structural, functional and physiological consequences of the modification and predict the importance in high CVD risk groups.

**Subjects/Methods:**

Major fractions of HDL, HDL2 and HDL3 were isolated from healthy human subjects and patients with type 2 diabetes and fractions modified by methylglyoxal and related dicarbonyl metabolites quantified. HDL2 and HDL3 were glycated by methylglyoxal to minimum extent *in vitro* and molecular, functional and physiological characteristics were determined. A one-compartment model of HDL plasma clearance was produced including formation and clearance of dicarbonyl-modified HDL.

**Results:**

HDL modified by methylglyoxal and related dicarbonyl metabolites accounted for 2.6% HDL and increased to 4.5% in patients with type 2 diabetes mellitus (T2DM). HDL2 and HDL3 were modified by methylglyoxal to similar extents *in vitro*. Methylglyoxal modification induced re-structuring of the HDL particles, decreasing stability and plasma half-life *in vivo*. It occurred at sites of apolipoprotein A-1 in HDL linked to membrane fusion, intramolecular bonding and ligand binding. Kinetic modelling of methylglyoxal modification of HDL predicted a negative correlation of plasma HDL-C with methylglyoxal-modified HDL. This was validated clinically. It also predicted that dicarbonyl modification produces 2–6% decrease in total plasma HDL and 5–13% decrease in functional HDL clinically.

**Conclusions:**

These results suggest that methylglyoxal modification of HDL accelerates its degradation and impairs its functionality *in vivo,* likely contributing to increased risk of CVD—particularly in high CVD risk groups.

## Introduction

The risk of cardiovascular disease (CVD) increases with age, diabetes and renal failure.^1,2^ Residual high risk of CVD in the general population suggests that CVD development is linked to risk factors unaddressed by current therapy. CVD mediated by arterial atherosclerosis has decreased plasma high-density lipoprotein cholesterol (HDL-C) as the risk factor. The major lipoprotein component of plasma HDL, apolipoprotein A-1 (ApoA1), correlates strongly with HDL-C and is more closely associated with anti-atherogenic protection.^3,4^ Impaired anti-atherogenic function of HDL independent of HDL-C is an emerging concept in the aetiology of CVD.^5^

**Figure 1:**
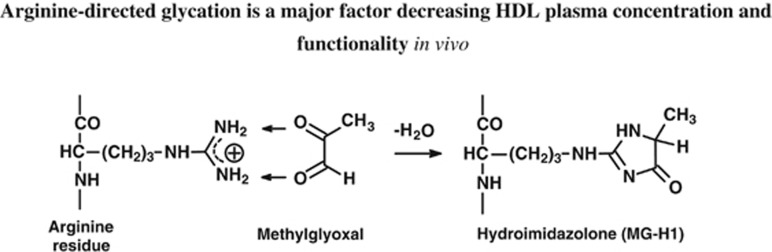
Reaction of methylglyoxal with arginine residues to form hydroimidazolone MG-H1.

In large prospective studies the risk of coronary heart disease was linked to ApoA1 and impaired glycemic control.^1^ Reviewing metabolic factors linked to dysglycemia, methylglyoxal—a reactive metabolite formed by the degradation of triosephosphates and metabolised by the glutathione-dependent glyoxalase system^6^—emerged as a potential mediator of HDL dysfunction. Plasma concentrations of methylglyoxal are increased by short-term and persistent increases in glucose concentration^7,8^—exacerbated by impairment of glyoxalase 1 expression and activity in oxidative stress, vascular inflammation and aging.^6,9^ Protein modification by methylglyoxal is relatively rapid and increases in aging, with further marked increases in diabetes and renal failure.^10, 11, 12^ Glycation of proteins by methylglyoxal is directed toward arginine residues, forming mainly the hydroimidazolone MG-H1—a quantitatively and functionally important advanced glycation endproduct (AGE) in physiological systems ^13^ (Figure 1). Herein we sought to characterise the extent of HDL modification by methylglyoxal and functional consequences in healthy people and predict the importance of this in high CVD risk groups, including patients with type 2 diabetes mellitus (T2DM).

## Materials and methods

### Healthy human subjects and patients with type 2 diabetes

Healthy, normolipidemic volunteers were recruited from friends and family members of the investigators and patients attending University Hospital of Coventry and Warwickshire, Coventry, UK. Recruitment criteria were as follows: absence of disease and dyslipidemia, age 18–70 years. Exclusion criteria were as follows: uncontrolled hypertension, CVD, renal or hepatic impairment, diabetes and other morbidities, severe excess alcohol consumption (>14/21 units per week for women/men), smoking, under pharmacological treatment affecting glucose and lipid metabolism or blood coagulation, and taking herbal remedies. Subject characteristics for healthy subjects were (mean±s.d., *n*=22) as follows: age 38.0±12.1 years; gender (M/F) 9/13; body mass index 23.9±3.0 kg m^−2^; total cholesterol 5.03±1.72 mM; LDL cholesterol 3.38±1.61 mM; HDL-C 1.11±0.60 mM; triglyceride 1.18±0.11 mM; and fasting glucose 5.17±0.43 mM. Patients with T2DM were recruited from those attending the Diabetes Clinic at University Hospital of Coventry and Warwickshire. Patient characteristics were (mean±s.d., *n*=7): age 60.1±7.3 years; gender 5/2; body mass index 26.0±6.1 kg m^−2^; total cholesterol 5.11±1.77 mM; LDL cholesterol 3.11±1.23 mM; HDL-C 1.26±0.34 mM; triglyceride 1.62±1.06 mM; fasting glucose 6.32±2.13 mM; and glycated haemoglobin HbA_1c_ 7.44±1.40%. Fasting venous blood samples were collected with EDTA anticoagulant with informed consent. Plasma was separated and HDL isolated immediately. Aliquots were stored at −80 °C for later analysis. The investigation conforms to the principles outlined in the Declaration of Helsinki. The studies with human subjects were approved by the Biomedical Research Ethics Committee, University of Warwick, Coventry, UK.

### HDL isolation, subfractionation, modification and characterisation

Plasma was subjected to sedimentation ultracentrifugation.^14,15^ very low-density lipoprotein and LDL were removed. Fractions corresponding to HDL2 (density, 1.125 g ml^−1^) and HDL3 (density, 1.210 g ml^−1^) were desalted and concentrated by washing with ice-cold argon-purged water by microspin ultrafiltration (100 kDa). Human recombinant ApoA1 and HDL subfractions (2.8 mg protein ml^−1^) were glycated by incubation with 1.5 mM methylglyoxal in phosphate-buffered saline (1.06 mM KH_2_PO_4_, 2.97 mM Na_2_HPO_4_ and 155 mM NaCl) containing 0.4 mM diethylenetriamine-penta-acetic acid, pH 7.4 and 37 °C, under argon for 6 h. Unmodified protein controls were incubated without methylglyoxal and processed similarly. Particle size distribution was assessed using 3–20% native polyacrylamide gel electrophoresis^16^ and electron microscopy. HDL stability was assessed by incubating HDL2 and HDL3 in 100 mM sodium phosphate buffer, pH 7.0 and 37 °C, for 48 h under argon and subsequent particle size estimation.^17^

### Cholesteryl ester transfer protein (CETP) activity

LDL was freshly isolated from plasma as described.^18^ HDL2 or MG_min_-HDL2 (0.3 μM) was incubated with LDL (1.0 μM) in lipoprotein-deficient human serum in the absence and presence of 1 mM 5,5′-dithiobis[2-nitrobenzoic acid] (DTNB), and 50 mM Tris/HCl, pH 7.4 and 37 °C. Samples (100 μl) were taken at baseline and after 48 h. An aliquot (50 μl) was treated with dextran sulphate (20 g l^−1^, 50 μl) and MgCl_2_ solution (2 M, 50 μl) and left at room temperature for 20 min to precipitate LDL. The samples were then centrifuged (4000 *g*, 15 min), the supernatant collected and total and free cholesterol were determined in HDL. Change in cholesteryl ester (CE) content from baseline provides an estimate of CETP activity *in situ.*^19^ DTNB is added to inhibit lecithin–cholesterol acyltransferase (LCAT) activity,^20^ which is required as LCAT would otherwise produce CE in HDL2 from cholesterol in the serum and interfere in the assay. DTNB does not inhibit CETP;^21^ there are functionally important thiols in CETP but they are not accessible to DTNB.^22^

### Analysis of glycation, oxidation and nitration adducts in ApoA1, HDL2 and HDL3

HDL (100 μg) derivatives were delipidated by precipitation with 20% trichloroacetic acid in saline and sequential extraction with acetone and diethyl ether. Residual protein was exhaustively hydrolysed enzymatically and glycated, oxidised and nitrated amino-acid content determined by stable isotopic dilution analysis liquid chromatography-tandem mass spectrometry.^23^

### Cell binding and metabolism studies

Human hepatoma HepG2 cells were incubated with MEM containing 20% lipoprotein-deficient serum for 24 h. Cells were seeded in 12-well plates, left to adhere for 48 h and were then used at 80% confluence. HDL preparations were radiolabelled with 125-iodine using pre-coated iodination tubes (Pierce Biotechnology, Rockford, IL, USA) according to the manufacturer's protocol and purified with gel filtration chromatography.^23^ [^125^I]-modified derivatives had specific activity in the range 1–2 × 10^3^ cpm ng^−1^. Cells were chilled to 4 °C for 60 min, washed three times, and binding of methylglyoxal-modified and control [^125^I]lipoprotein were determined in the absence (total binding) or presence (nonspecific binding) of a 10-fold excess of unlabelled HDL over 60 min at 4 °C. For HDL metabolism studies, cells were washed three times and then incubated at 37 °C for 5, 10 15, 30, 60 and 90 min in the presence of [^125^I]-labelled HDL derivative. HDL metabolism was assessed by internalised radioactivity after washing cells twice with ice-cold MEM and incubating at 4 °C for 90 min.

### Plasma clearance and organ retention of HDL *in vivo*

Thirty-two adult male Sprague–Dawley rats (Charles River, UK) were divided into four groups and received an intravenous dose of [^125^I]-labelled methylglyoxal-modified and control HDL2 and HDL3 (200 μg lipoprotein, 65 kBq) in phosphate-buffered saline. Blood samples (0.1 ml) were taken 5, 10, 15, 30 and 45 min post dosing, and at 60 min killed using terminal anaesthesia induced by injection of sodium pentobarbital in the tail vein. Blood, kidney and liver samples were collected. Time course plasma activity data were used to deduce HDL clearance rates and radioactivity in the kidney, liver and blood used to deduce HDL tissue/blood partitioning. The investigation conforms to the Directive 2010/63/EU of the European Parliament and was approved by the local Biological Ethics Committee and performed under UK Home Office Project license no PPL40/3260.

### Proteomic analysis of ApoA1, HDL2 and HDL3

Delipidated lipoproteins (100 μg lipoprotein) were reduced, alkylated and then digested with trypsin. ApoA1 and MG_min_-ApoA1 were digested similarly. Digests were analysed using nanoflow liquid chromatography-ion trap mass spectrometry with alternating collision-induced dissociation and electron transfer dissociation peptide fragmentation and peptides with and without MG-H1 modification identified and sequenced. Identification and quantification of sites of methylglyoxal modification in Apo-A1 were made as previously described.^23^ Delipidated lipoprotein (100 μg in 100 μl phosphate-buffered saline) was reduced by incubation with dithiothreitol (12.5 mM, 2 μl) at 37 °C in the dark for 30 min. This step was omitted for Apo-A1 and MG_min_-ApoA1 that lack cysteine residues. Iodoacetamide (24.4 mM, 2 μl) was then added and incubated at 37 °C in the dark for a further 30 min. Residual iodoacetamide was then quenched by addition of dithiothreitol (12.5 mM, 2 μl) and incubated at 37 °C in the dark for 30 min. TPCK-treated trypsin (1 mg ml^−1^, 4 μl) was then added at an enzyme-to-substrate ratio of 1:25 (w/w) in 1 mM CaCl_2_/100 mM NH_4_HCO_3_ buffer, pH 8.5, and the sample was incubated at 37 °C for 10 h in the dark. The reaction was stopped using 1% acetic acid and the pH was adjusted to pH 3. The sample was lyophilised to dryness and reconstituting in water; final concentration was 1 μg lipoprotein equivalent per μl. Digests were analysed using nanoflow liquid chromatography-ion trap mass spectrometry with alternating CID and electron transfer dissociation peptide fragmentation: EASY-nLC interface, flow rate of 300 nl min^−1^, with fluoranthene electron/proton donor-AmaZon mass spectrometer (Bruker Daltonics, Bremen, Germany). CID fragmentation was controlled with SmartFrag (Bruker Daltonics, Bremen, Germany). Electron transfer dissociation fragmentation was performed with 500 000 counts of fluoranthene present in the trap for 100 ms, and Smart Decomposition set to auto. Fragmented peptide ion series, CID – *b* and *y* ions, and CID – *z* and *c* ions, were analysed for peptide sequencing and thereby identification and sequence location. MG-H1 residues are identified by characteristic mass increment of 54 Da to the arginine residue-modified and -related ion series. Spectra were processed using DataAnalysis (Bruker Daltonics) and the resulting peak lists were subjected to database searching using Mascot (Matrix Sciences, London, UK). Good peptide fragmentation was observed and peptide Mascot scores were >15. Peptides were present throughout the 28-kDa sequence of the protein. Quantitation of modification was by the label-free proteomics technique.^24^ Unmodified arginine residue-containing peptide ion responses were normalised to the *C*-terminal peptide ion response as internal standard. The mean normalised peptide response ratio in methylglyoxal-modified samples is compared with that of controls to deduce the loss of arginine residue-containing peptide by methylglyoxal modification. The mean coefficient of variation of normalised peptide responses was 33%.

### Molecular modelling

MG-H1 residues were built onto ApoA1 at identified hotspot modification sites in the refined structural models of HDL, the trefoil model^25^ and the *N-*terminal 1–43 residues of an *N*-terminally truncated apoA1 mutant.^26^

### One-compartment kinetic model of HDL catabolism and dicarbonyl glycation in plasma

Kinetics and pool size of human HDL and changes with increased plasma dicarbonyl concentration were modelled in COPASI^27^ using estimated parameters in stable isotope studies.^28^

### Statistical analysis

Data are mean±s.d. for parametric data and the median (upper–lower quartile) for non-parametric data. Significance of difference of the mean changes was assessed by Student's *t*-test and of the median changes by Mann–Whitney *U-*test. For HDL plasma clearance studies, radioactive counts were fitted to a single exponential and plasma half-life deduced.

## Results

### Modification of HDL2 and HDL3 by methylglyoxal *in vivo* and *in vitro*

Methylglyoxal-derived MG-H1 was a major adduct of lipoprotein of HDL2 and HDL3 isolated from healthy human subjects. MG-H1 content (mol%) was: HDL2 1.0% and HDL3 0.8%. HDL2 and HDL3 were also modified by similar hydroimidazolone AGEs formed by glycation with related dicarbonyl metabolites, glyoxal and 3-deoxyglucosone. The total dicarbonyl-modified HDL (DC-HDL) derivatives accounted for 2.8% HDL2, 2.4% HDL3 and 2.6% total HDL in healthy people. In a pilot study of patients with T2DM we found that DC-HDL accounted for 5.6% HDL2, 3.3% HDL3 and 4.5% total HDL (*P*<0.05; Table 1).

ApoA1, HDL2 and HDL3 were prepared with similar low extent of modification by methylglyoxal as found *in vivo*. These preparations, MG_min_-ApoA1, MG_min_-HDL2 and MG_min_-HDL3, respectively, were made by incubating recombinant ApoA1 and HDL subfractions with methylglyoxal *in vitro*. Preparations had 1.8–2.6 molar equivalents of MG-H1 and trace amounts (⩽0.6%) of other methylglyoxal-derived glycation adducts, N_ɛ_(1-carboxyethyl) lysine and imidazolium crosslink (MOLD; Supplementary Table S1). HDL2 and HDL3 contents of oxidation adduct methionine sulfoxide and nitration adduct 3-nitrotyrosine were low (0.08 and <0.004 mol%, respectively). Both were unchanged by methylglyoxal modification. These derivatives were employed to study changes in biophysical and biological characteristics imposed by minimal glycation with methylglyoxal.

### Modification of HDL2 and HDL3 by methylglyoxal *in vitro* decreases particle size, stability and functionality

**Figure 2:**
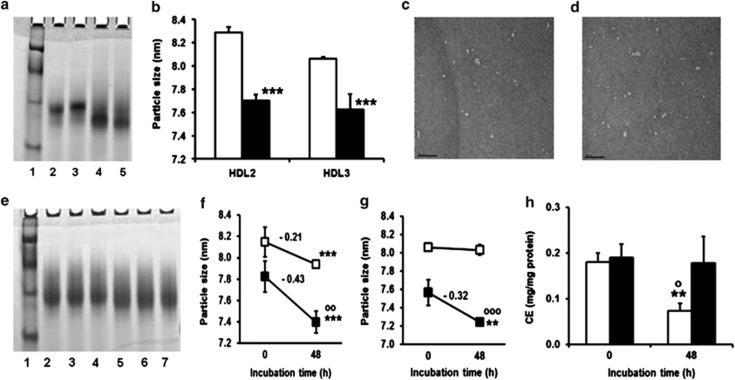
Methylglyoxal modified HDL: decreased particle size and stability. (**a**) HDL particle size assessed by native gel electrophoresis. Data are mean±s.d. (*n*=4). Significance: ***P<0.001. (**b**) HDL2 and HDL3 modified by methylglyoxal. Lane Key: (1) molecular diameter markers (thyroglobulin, 17.0 nm, ferritin 12.2 nm, catalase 9.7 nm, lactate dehydrogenase 8.1 nm and albumin 7.1 nm); (2) MG_min_-HDL2; (3) HDL2; (4) HDL3; and (5) MG_min_-HDL3. (**c** and **d**) Electron micrographs of HDL2 and MG_min_-HDL2. Magnification: 25,000. (**e**) Decreased stability of HDL at pH 7.4 and 37 °C after modification by methylglyoxal assessed by decrease in particle size over incubation for 48 h. Typical native gel electrophoresis scans: HDL2 and MG_min_-HDL2 incubated in 100 mM sodium phosphate buffer pH 7.0 at 37°C for 48 h. Lane key: 1, molecular diameter markers; 2–4 HDL2 control; 5–7 MG_min_-HDL2. (**f**, **g**) Effect on stability of HDL2 and HDL3, respectively. Key - unmodified HDL (-□-□-) and MG_min_-HDL (-▪-▪-). Data are mean±s.d. (*n*=3–6). Significance: **P<0.01 and ***P<0.001 with respect to baseline; ^oo^*P*<0.01 and ^ooo^*P*<0.001 for change from baseline with respect to unmodified control. (**h**) Effect of methylglyoxal modification of HDL2 on cholesteryl ester transfer to LDL. Key: hollow bar, unmodified HDL2; solid bar, MG_min_-HDL2. Significance: **P<0.01 with respect to baseline; ^o^*P*<0.05 with respect to unmodified control at 48 h. Data are mean±s.d. (*n*=3).

The effect of modification on HDL particle size and stability was investigated by gradient gel electrophoresis. Methylglyoxal modification produced decreased particle size of HDL2 and HDL3 (Figure 2a, specimen gel scan) and Figure 2b (deduced particle size). This was corroborated with particle size measurement using transmission electron microscopy with expected, slightly higher absolute size estimate.^29^ HDL particle diameter by this method is typically larger than determined by gradient gel electrophoresis and for HDL2 gave: control, 10.4±1.1 nm, and MG_min_-HDL2, 8.7±0.9 nm (−16%, *P*<0.001; *n*=50; Figure 2c and d). The size of MG_min_-HDL2 is typical of small dense, dysfunctional HDL *in vivo* associated with increased risk of CVD.^30^ Incubation of MG_min_-HDL2 and MG_min_-HDL3 under physiological conditions produced an accelerated decrease in particle size indicative of decreased particle stability (Figure 2e; specimen gel scan) and Figures 2f and g (deduced particle sizes).

Interaction of CETP with HDL2 promotes the exchange of CE and TG with LDL.^5^ We investigated the effect of methylglyoxal modification of HDL on CE transfer by incubating HDL2 and MG_min_-HDL2 with LDL *in vitro* and measuring CE transfer. We found that CE transfer was inhibited completely for MG_min_-HDL2 (Figure 2h).

**Figure 3:**
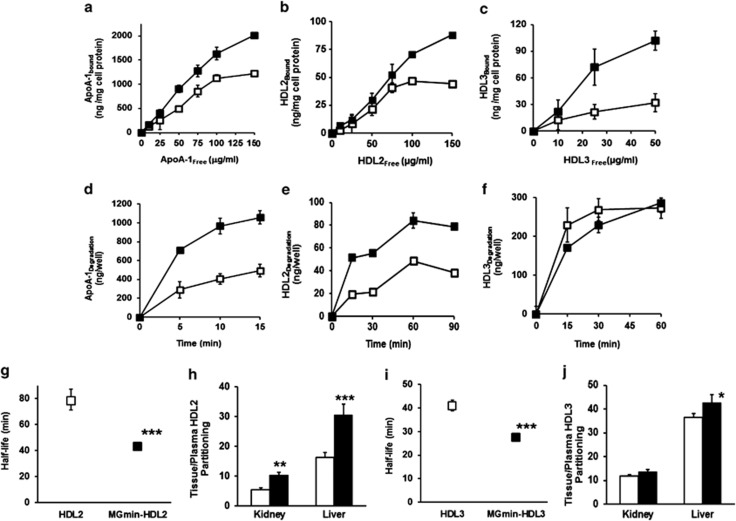
Increased hepatocyte-like cell binding and degradation *in vitro* and increased plasma clearance *in vivo* of HDL modified by methylglyoxal. Hepatocyte-like HepG2 cells *in vitro* cell surface binding—(**a**) ApoA1, (**b**) HDL2 and (**c**) HDL3; metabolism—(**d**) ApoA1, (**e**) HDL2 and (**f**) HDL3. Data are mean±s.d. (*n*=4). Plasma clearance and partitioning from plasma to the kidney and liver of HDL in rats: plasma clearance curves (with exponential fits), half-lives and tissue partitioning - HDL2 - (**g**, **h**) and HDL3 (**i**, **j**). Plasma clearance data per animal were normalised to total counts in blood at 5 min post injection and exponential decrease over the following 55 min deduced. Data are mean±s.e.m. (*n*=8). Key: □-□, control (unmodified) and ▪-▪, methylglyoxal modified. Significance: *P<0.05, **P<0.01 and ***P<0.001.

A key physiological function of HDL is reverse cholesterol transport, delivering cholesterol and triglycerides to the liver from peripheral tissues.^31^ Binding of HDL to hepatocyte plasma membrane occurs via the hepatic scavenger class B receptor, types I and type II.^32,33^ We studied the binding of methylglyoxal modified ApoA1, HDL2 and HDL3 to human hepatocyte-like HepG2 cells *in vitro*. Cell binding by HepG2 cells of all methylglyoxal-modified forms was increased compared with unmodified controls (Figures 3a–c). Metabolism was also increased of modified forms except for MG_min_-HDL3 (Figures 3d–f).

The plasma half-life and tissue partitioning of HDL derivatives in rats was studied. For unmodified HDL2 and HDL3 we found similar plasma half-life as in previous studies:^34^ the mean plasma half-life of HDL2 and HDL3 was 78.3 and 41.0 min, respectively. The plasma half-life of MG_min_-HDL2 was decreased to 42.8 min (−45%) and that of MG_min_-HDL3 decreased to 27.5 min (−33%). The liver is the major site of catabolism of human HDL in rats with ApoA1 peptide excretion via the kidney in urine.^35^ There was increased partitioning of MG_min_-HDL2 and MG_min_-HDL3 from plasma into the liver and partitioning of MG_min_-HDL2 from plasma to the kidney (Figures 3g–j).

### Hotspots of HDL modification by methylglyoxal are functional sites of ApoA1

**Figure 4:**
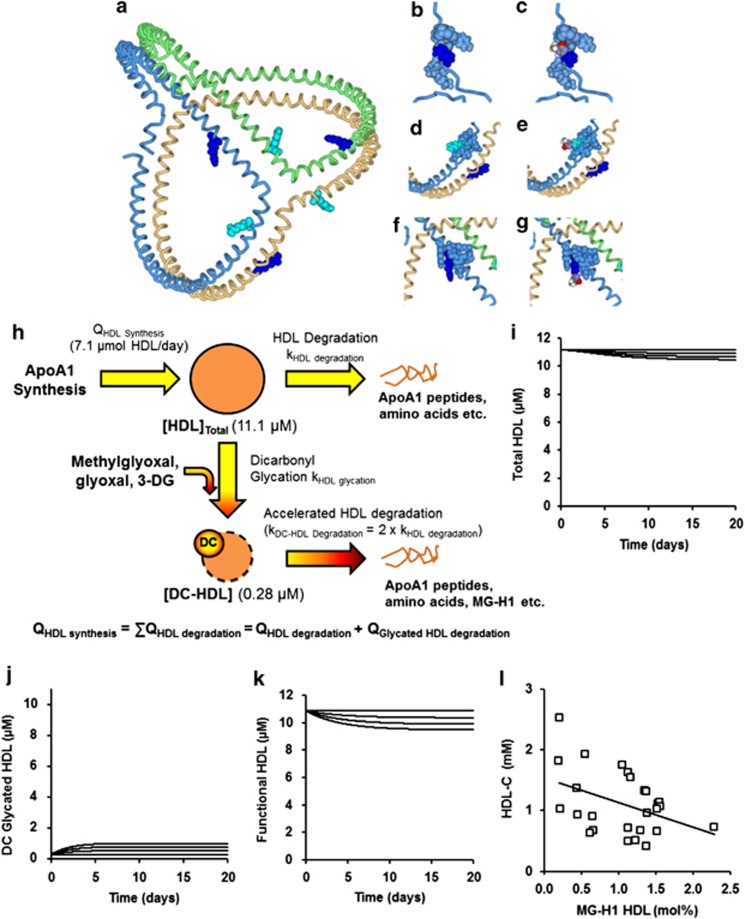
Molecular sites of modification and metabolic modelling of methylglyoxal-modified HDL A. Structural basis of functional change of methylglyoxal-modified HDL. Molecular model of human ApoA1 residues 40–243. Schematic representation: trefoil structure of trimeric ApoA1 with colour-coded peptide chains and hotspot methylglyoxal, glycation sites on each chain: R123 (cyan) in helix 5 and R149 (dark blue) in helix 6 either sides of hinge at residue 133. (**b**, **c**) R27 and MG-H1–27, respectively, in the *N*-terminal domain 1–43 (ref. 26). (**d**, **e**) R123 and MG-H1–123 in helix 5. (**f**, **g**) R149 and MG-H1–149 in helix 6. Hydroimidazolone rings are conventional element colour-coded. (**h**) One-compartment modelling of the effect of dicarbonyl glycation on plasma HDL. Panels (**i**–**k**) show relaxation from the steady state of healthy subjects (time zero) to new steady states of two-, three- and fourfold increased dicarbonyl concentration, (**i**) decreasing concentration series of total HDL, (**j**) increasing concentration series of DC-HDL and (**k**) decreasing concentration series of functional HDL. Parameters used: (HDL), 11.1 μM; (DC-HDL), 0.28 μM; (Dicarbonyl), 0.277 μM (and x 2, 3 and 4). Rate of HDL synthesis, 2.33 μmol per day; k_HDL degradation_, 0.204 per day; k_HDLglycation_, 0.038 μmol^−1^per day (deduced from the rates of glycation in preparation of HDL2 and HDL3 modified minimally by methylglyoxal); and k_DC-HDLdegradation_, 0.408 per day (assumed 2 x k_HDL degradation_). (**l**) Negative association of plasma HDL-C to MG-H1 content of HDL.

The current accepted molecular model for the structure of ApoA1 in HDL is the trefoil structure.^36^ R123 and R149 are shown on the trefoil peptide backbone structure (Figure 4a). In replicate digests methylglyoxal modification was detected in tryptic peptides derived from MG_min_-ApoA1, MG_min_-HDL2 and MG_min_-HDL3. MG-H1 residues were detected at R27 in peptide DSGR_MG-H1_DYVSQFEGSALGK (residues 24–40) and R123 in peptide VEPLR_MG-H1_AELQEGAR (residues 119–131) of Apo-A1, HDL2 and HDL3, and at R149 in peptide LSPLGEEMR_MG-H1_DR (residues 141–151) of ApoA1 and HDL2 (Table 2 and Supplementary Figures S1–S3). All arginine-containing peptides were detected; only those indicated above had significant modification by methylglyoxal. Summing all modifications detected, we located 78% total modification in MG-ApoA1, 53% in MG-HDL2 and 46% in MG-HDL3. From the statistical power of the label-free proteomic analysis, the remaining modification is expected to be distributed on multiple other sites at <32% modification. Hence, it is unlikely that other sites of major methylglyoxal modification have been overlooked.

MG-H1 was built on to structural models of HDL at R27, R123 and R149 and changes of bonding interactions identified. The *N*-terminal domain containing R27 was not part of the proposed trefoil structural model;^36^ rather the structure assumed was that based on the crystal structure of an *N*-terminally truncated apoA1 mutant.^26^ In unmodified HDL, R27 had electrostatic salt bridge interaction with D29: predicted bonding interaction lengths of the terminal N and O atoms are N^η1^(R27)-O^δ1^(D29) and N^η2^(R27)-O^δ2^(D29) of 1.70 and 1.64 Å, respectively. With methylglyoxal modification, MG-H1–27 lost salt bridge interaction (MG-H1 side chain has no charge) and had weaker hydrogen bonding with D24: N^δ^(R27)-O^δ^(D24), bond length 1.94 Å (Figures 4b and c). R123 is in helix 5 and has an ion-pair interaction with E120 of helix 4—bonding interaction length N^η1^(R123)-O^δ^(E120) 4.34 Å,^37^ which is lost with MG-H1–123 formation (Figures 4d and e). R149 in helix 6 has no predicted change of intramolecular binding on modification to MG-H1–149 (Figures 4f and g).

### Kinetic modelling of the effect of glycation by methylglyoxal on plasma clearance of HDL in human subjects

A one-compartment model of HDL influx and clearance from plasma in human subjects was produced defined by published estimates of values of ApoA1 synthesis (14 mg kg^−1^per day)^28^ and HDL half-life (4.47 days).^38^ We introduced a new kinetic pathway of dicarbonyl modification of HDL producing DC-HDL with a twofold increased plasma clearance—as found in rat studies. HDL kinetics were computed for two- to fourfold increase in dicarbonyl concentration typical of old age, diabetes and renal failure.^6,9,39^ The kinetic model predicted a DC-HDL concentration of 5–9% total HDL, a decrease of total plasma HDL by 2–6% and a decrease of functional HDL (total HDL minus DC-HDL) of 5–13%. The model predicted an inverse association of plasma HDL concentration to the extent of modification of HDL by dicarbonyl-derived hydroimidazolone AGEs. Returning to the AGE content of HDL2 and HDL3 isolated from healthy human subjects, there was indeed a negative correlation of plasma HDL-C concentration with MG-H1-modified HDL in healthy people; *r*=−0.42, *P*<0.05 (*n*=22, Pearson).

## Discussion

This study identified a novel endogenous modification converting HDL to a destabilised and functionally impaired variant—methylglyoxal-derived hydroimidazolone MG-H1. Biophysical studies of HDL2 and HDL3 modified by methylglyoxal *in vitro* herein showed decreased HDL particle size consistent with structural contraction and increased density. Methylglyoxal-modified HDL2 and HDL3 had an accelerated decrease in particle size during incubation under physiological conditions indicative of decreased particle stability. This suggests that low physiological extents of modification of HDL by methylglyoxal produce structural alterations that destabilise HDL2 and HDL3 particles. Protein modifications of HDL were reviewed previously.^40^ MG and glyoxal adducts were determined by immunoassay without robust and absolute quantitation. The increase in MG-derived adducts of HDL reported in patients with diabetes (type not specified) was *ca.* 50%,^41^ which is in reasonable agreement with the 70% increase in MG-H1 content of total HDL in patients with T2DM found herein, with respect to healthy controls.

Studies of HDL2 and HDL3 plasma clearance *in vivo* indicated that methylglyoxal modification increases plasma clearance and hepatic metabolism of HDL lipoprotein *in vivo*, a characteristic associated with decreased HDL particle size.^42^ Increased clearance of methylglyoxal-modified HDL will tend to counter accumulation of methylglyoxal-modified HDL, producing a less than proportionate increase in steady state of methylglyoxal-modified HDL with increased plasma methylglyoxal. This may explain why the increase in methylglyoxal-modified HDL was relatively low in patients with T2DM; *cf.* increase in methylglyoxal modification in total plasma protein of diabetes of *ca*. 3-fold.^11^ Decreased stability of methylglyoxal-modified HDL may also lead to increased shedding of lipoproteins and contribute to increased renal catabolism of HDL.^43^ This is also a feature of modification by similar physiological dicarbonyl metabolites: glyoxal and 3-deoxyglucosone, formed by lipid peroxidation, fructosamine degradation and other sources.^11^ Supporting evidence that methylglyoxal disturbs HDL metabolism came from administration of methylglyoxal (60 mg kg^−1^) into rats that decreased plasma HDL by 42%.^44^ Destabilization and increased clearance of HDL by methylglyoxal modification likely contributed this effect.

Proteomic peptide mapping studies of methylglyoxal-modified ApoA1, HDL2 and HDL3 showed that glycation occurs on ApoA1 at R27, R123 and R149. Molecular dynamics modelling of the N terminus of ApoA1 identified a cluster of nine interhelical salt bridges that is highly conserved.^45^ This domain is ‘sticky' in that it latches together with salt bridges until sufficient interaction with phospholipid relaxes them and facilitates particle membrane fusion. With MG-H1 modification at R27 particle membrane fusion may be facilitated leading to increased hepatic and renal internalisation and degradation. HDL2 is the major form by which ApoA1 is degraded *in vivo.*^46^ R123 of ApoA1 enhances HDL structural stability by salt bridge interhelical bonding with E120. Modification to MG-H1 in HDL2 and HDL3 likely leads to axial twist of the open trefoil cradle structure, increasing particle density and decreasing particle size. R149 is part of a triad of residues with R153 and R160 that interact electrostatically with LCAT, paraoxonase-1 and oxidised phospholipid.^45,47^ These interactions may be weakened in HDL2 with modification to MG-H1.

Other protein modifications of ApoA1—particularly methionine oxidation and tyrosine nitration^48,49^—have been implicated in dysfunctional HDL and risk of CVD. Methionine sulfoxide and 3-nitrotyrosine contents of HDL2 and HDL3 were quantitatively low, which may indicate limited physiological significance, at least in the healthy population. FL and N_ɛ_-carboxymethyl-lysine residue contents of HDL2 and HDL3 were reported previously.^50^ Glycation of HDL by glucose-forming FL mainly on K239 in the *C*-terminal domain has been studied previously but did not change plasma clearance.^51,52^ The *C*-terminal domain is not involved in structural stability of the HDL particle and is thought to interact electrostatically with the *N*-terminal domain.^53^ K239 retains side chain-positive charge and electrostatic interactions when glycated by glucose. Increased clearance and degradation of HDL is rather linked to arginine-directed modification by methylglyoxal. HDL modified by methylglyoxal has been prepared *in vitro* previously where preparations had 42–100 modifications per HDL particle (arginine and lysine) to study effect on LCAT activation.^54^ This extent of HDL modification has limited physiological relevance being markedly higher than the one modification per particle found on 2.6% HDL *in vivo* reported herein.

Methylglyoxal modification of HDL2 inhibited CETP activity. Recent structural studies suggests that CETP interacts with the lipid core of HDL:^55^ the *ca.* 10 × 3 nm banana shape of CETP was found to penetrate into the hydrophobic core of 8.5- to 10-nm diameter HDL particles, through the ApoA1 trefoil structure at the particle surface.^25^ Methylglyoxal-induced contraction of the trefoil framework may restrict the approach of CETP to the hydrophobic core and decrease CETP activity. In contrast, CETP inhibitors such as anacetrapib, torcetrapib and dalcetrapib are thought to inhibit CETP by increasing its affinity for HDL.^56^ Inhibition of CETP was thought to be beneficial but this may require revision.^5^ Blocking CE-TG exchange may lead to increased TG-rich, small dense LDL with increased atherogenicity. Methylglyoxal modification also increases the atherogenicity of LDL directly by decreasing LDL particle size and increasing density, aggregation, binding to arterial proteoglycans and partitioning on the aortal wall *in vivo.*^23^ Functional changes and atherogenicity of HDL subpopulations remain to be fully characterised and their role in atherogenicity and anti-atherogenicity understood.^57^

To assess the physiological significance of dicarbonyl modification of HDL2 and HDL3 by *ca.* 2.6% found herein in healthy people—particularly in relation to the reported 20 and 37% decrease in half-life of HDL in T2DM and renal failure^28,58^—we constructed a one-compartment model of HDL release into and clearance from the plasma, assuming that DC-HDL is cleared twice as fast as unmodified HDL—as found in the preclinical *in vivo* studies herein. From model predictions dicarbonyl modification contributes significantly to decreased half-life and dysfunction of HDL, and through this to increased CVD risk in high-risk populations such as elderly and patients with T2DM and renal failure. A two- to fourfold increase in plasma dicarbonyl concentration predicted a 2–6% decrease in plasma total HDL. As HDL (ApoA1) correlates strongly with HDL-C (*r*=0.83),^3^ this translates to a predicted *ca.* 3–9% increased risk of CVD.^59^ The model also predicted a greater decrease in functional HDL (4.5–12.5%). This may suggest a greater overall impact on CVD risk, contingent on further evidence of importance of HDL dysfunction on CVD risk. The model was validated by experimental confirmation of the predicted negative link of plasma HDL (and thereby HDL-C) concentration to the fraction of HDL modified by methylglyoxal (mostly MG-H1 adduct residues) in healthy people. From the square of the correlation coefficient, *r*=−0.42, the relationship implies that 18% of HDL-C variation is linked to methylglyoxal modification—a significant component of variation unaddressed by current therapy. Dicarbonyl glycation, therefore, likely contributes significantly to decreased plasma HDL-C, dysfunctional HDL and increased CVD risk in the general population.

Limitations of this study are the pilot nature of the clinical study. The two major HDL subfractions, HDL2 and HDL3, were studied, whereas additional further subfraction may be considered in future studies.^60^

## Figures and Tables

**Table 1:** Protein glycation, oxidation and nitration adduct content of HDL2 and HDL3 of healthy people and patients with type 2 diabetes

*Modification type*	*Adduct*	*Healthy controls (*n=*22)*		*T2DM (*n=*7)*	
		*HDL2*	*HDL3*	*HDL2*	*HDL3*
Methylglyoxal-derived AGE	MG-H1	1.00±0.54	0.82±0.56	1.60±0.42*	1.57±0.49**
	CEL	0.09 (0.05–0.15)	0.10 (0.06–0.14)	0.19 (0.11–0.33)*	0.12 (0.10–0.16)
	MOLD	0.015 (0.009–0.038)	0.043 (0.014–0.109)	0.025 (0.005–0.137)	0.028 (0.019–0.046)
Other AGE adducts/variables	CML	0.38±0.20	0.51±0.37	0.41±0.19	0.65±0.27
	Total arg-derived AGE	2.31±0.54	1.44 (0.82–3.42)	5.24±1.83***	3.10 (2.39–5.19)*
	Total dicarbonyl adducts	2.82±1.59	2.42±1.89	5.56±1.90**	3.25±1.79*
Early glycation adduct	FL	3.02 (1.58–8.05)	5.26 (2.95–6.39)	1.88 (1.55–1.94)	3.54 (2.19–5.42)
Oxidation marker	MetSO	0.35±0.18	0.21 (0.12–0.40)	0.41±0.11	0.51 (0.46–0.52)*
Nitration marker	3-NT	0.010 (0.006–0.031)	0.009 (0.005–0.031)	0.009 (0.007–0.011)	0.007 (0.0060)

Abbreviations: AGE, advanced glycation endproduct; CEL, N_ɛ_(1-carboxyethyl) lysine; CML, N_ɛ_-carboxymethyl-lysine; MetSO, methionine sulfoxide; 3-NT, 3-nitrotyrosine.

Data are adduct contents (mol%, mean±s.d. or median (lower–upper quartile). Significance: *P<0.5, **P<0.01 and ***P<0.001 with respect to healthy subjects. Total arg-derived AGE is the sum of arginine-derived AGEs: MG-H1 and hydroimidazolones derived from glyoxal and 3-deoxyglucosone (G-H1 and 3DG-H, respectively) and N_ω_-carboxymethyl-arginine (CMA). Individual G-H1, 3DG-H and CMA estimates are not shown for brevity. DC-HDL: arginine-derived AGEs+N_ɛ_(1-carboxyethyl) lysine+ MOLD. N_ɛ_-carboxymethyl-lysine is not included as it is derived mostly from the oxidative degradation of FL. Adduct content in lipoprotein-exhaustive digests was deduced as mol/mol amino acid modified by quantitation of analyte and related amino-acid contents—for example, for MG-H1, mol MG-H1/mol arg. Analyte content was then converted to mol% HDL by multiplying molar content of amino acid modified in HDL x 100—for example, for MG-H1 multiplied mol arg/mol HDL. Amino-acid content of HDL2 and HDL3 (mol/mol HDL) was deduced from the major protein composition (ApoA1, ApoA2, Apo C2 and transferrin):^14^ HDL2–arg 66.4, lys 121.7, met 18.3 and tyr 48.2; HDL3–arg 65.2, lys 114.3, met 16.2 and tyr 42.8. HDL2 and HDL3 mol fractions in healthy controls was 0.50±0.12 for both.

**Table 2:** Peptide mapping of ApoA1, HDL2 and HDL3 modified minimally by methylglyoxal *in vitro*

*Modified arginine*	*Peptide*	*Peptide detected*	*Sequence*	*Charge z+*	*Molecular ion*	*Extent of modification (%)*		
						*ApoA1*	*HDL2*	*HDL3*
27	24–40	Unmodified	DSGRDYVSQFEGSALGK	3	606.07 (M+3)/*z*	—	—	—
		Modified	DSGR_MG-H1_DYVSQFEGSALGK	3	624.03 (M+3)/*z*	59±35*	41±10*	32±12*
123	119–131	Unmodified	VEPLRAELQEGAR	3	489.99 (M+3)/*z*	—	—	—
		Modified	VEPLR_MG-H1_AELQEGAR	3	507.93 (M+3)/*z*	57±35*	52±13**	49±11**
149	141–151	Unmodified	LSPLGEEMRDR	3	434.97 (M+3)/*z*	—	—	—
		Modified	LSPLGEEMR_MG-H1_DR	3	452.95 (M+3)/*z*	84±12**	30±5*	—
				Modification detected (mol%)		200	123	81
				Total modification (mol%)		256	233	177

Abbreviations: ApoA1, apolipoprotein A-1; HDL, high-density lipoprotein;

Data are mean±s.d. Significance: *P<0.05 and **P<0.01. Modification detected is the sum of the extents of all MG modifications found. Total modification is the MG-H1 content reported in Table 2, given here in mol%.
