# Supplementary material for: Arginine-directed glycation and decreased HDL plasma concentration and functionality
Source: Nutr Diabetes. 2014 Sep 1;4(9):e134–. doi: 10.1038/nutd.2014.31 (PMC4183972; doi:10.1038/nutd.2014.31)
Supplement: Supplementary Material [file nutd201431x1.doc]

**SUPPLEMENTARY MATERIAL**

**SUPPLEMENTARY TABLE**

**Table S1.** Protein glycation adduct residues in human ApoA1, HDL2 and HDL3 modified minimally by methylglyoxal *in vitro*.

| Protein/ particle |  | Analyte (mol/mol ApoA1) | | |
| --- | --- | --- | --- | --- |
|  | n | MG-H1 | CEL | MOLD |
| ApoA1 | 3 | 0.003 ± 0.001 | 0.0013 ± 0.0003 | 0.0001 ± 0.0001 |
| MG_min_-ApoA1 | 3 | 2.558 ± 0.185*** | 0.0017 ± 0.0004 | 0.0008 ± 0.0001*** |
| HDL2 | 7 | 0.006 ± 0.005 | 0.0003 ± 0.0001 | 0.0002 ± 0.0001 |
| MG_min_-HDL2 | 7 | 2.327 ± 0.541*** | 0.0030 ± 0.0014*** | 0.0018 ± 0.0005*** |
| HDL3 | 3 | 0.009 ± 0.007 | 0.0004 ± 0.0003 | 0.0001 ± 0.0001 |
| MG_min_-HDL3 | 4 | 1.772 ± 0.288*** | 0.0043 ± 0.0003*** | 0.0042 ± 0.0005*** |

Analyte mol fraction (mol/mol ApoA1) was deduced as described in Table 1. Significance: ***, P<0.001. MG_min_-ApoA1, MG_min_-HDL2 and MG_min_-HDL3 were prepared by incubating ApoA1 and HDL2 and HDL3 subfractions (2.8 mg protein/ml) with 1.5 mM methylglyoxal in PBS containing 0.4 mM DETAPAC for 6 h at pH 7.4 and 37^o^C under anaerobic conditions. Unmodified protein controls were incubated without methylglyoxal and processed similarly. After incubation excess methylglyoxal was removed by 4 cycles of concentration and dilution with PBS (500 to 50 μl), pH 7.4 and 4^o^C, in microspin filters (molecular mass cut-off 12 kDa).

**SUPPLEMENTARY FIGURES**

Cont’d below

**Fig. S1.** Location of sites of hotspot glycation by methylglyoxal in apolipoprotein A1 by ETD ion trap mass spectrometry - glycation site at R27. Mass spectrum of z+1 and c ion series of amino acids 24 - 40. Methylglyoxal modification on R*. Biotools score 156.

**
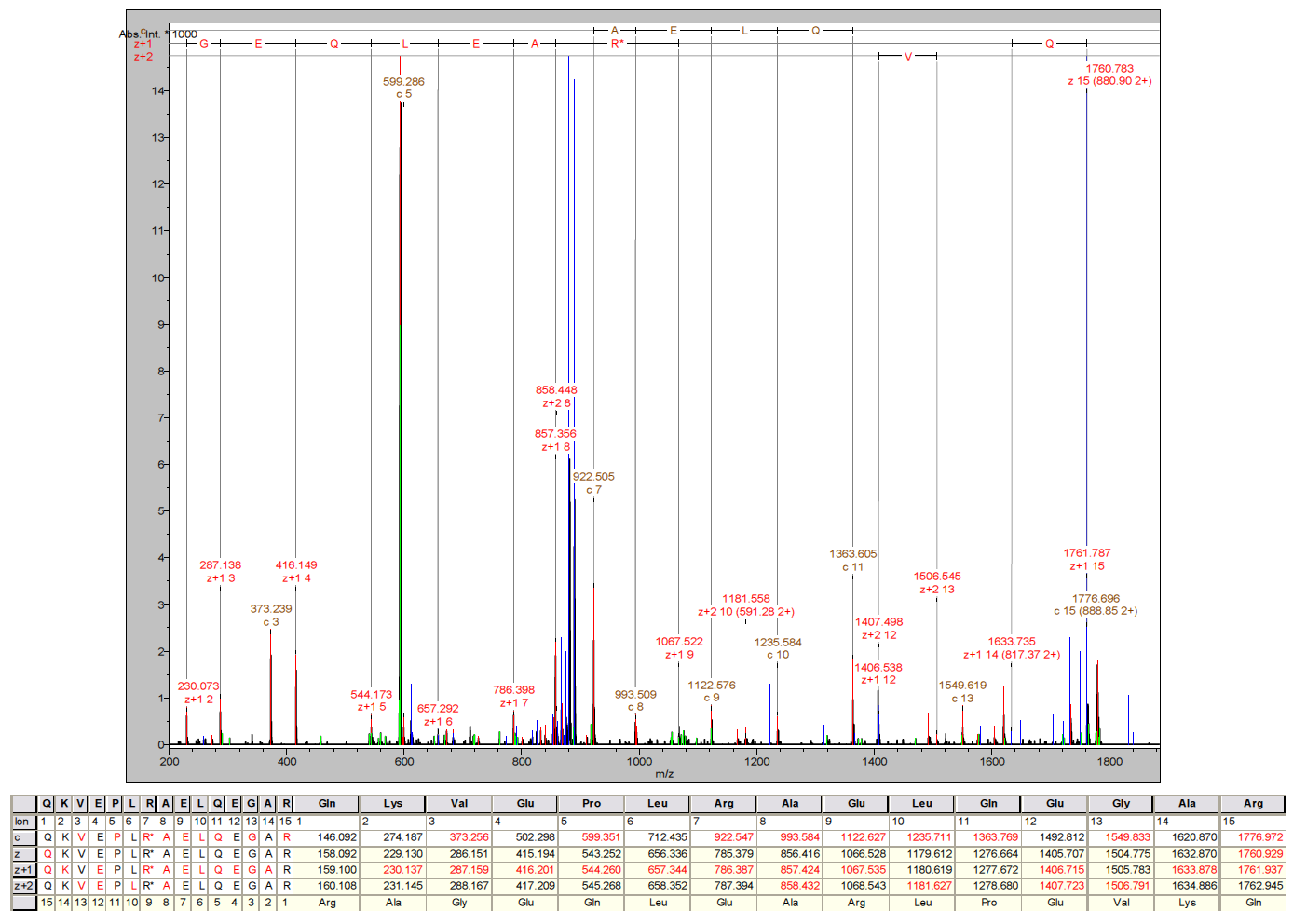
**

**Fig. S2.** Location of sites of hotspot glycation by methylglyoxal in apolipoprotein A1 by ETD ion trap mass spectrometry - glycation site at R123. Mass spectrum of z+1 and c ion series of amino acids 117 – 131. Methylglyoxal modification on R*. Biotools score 299.

**
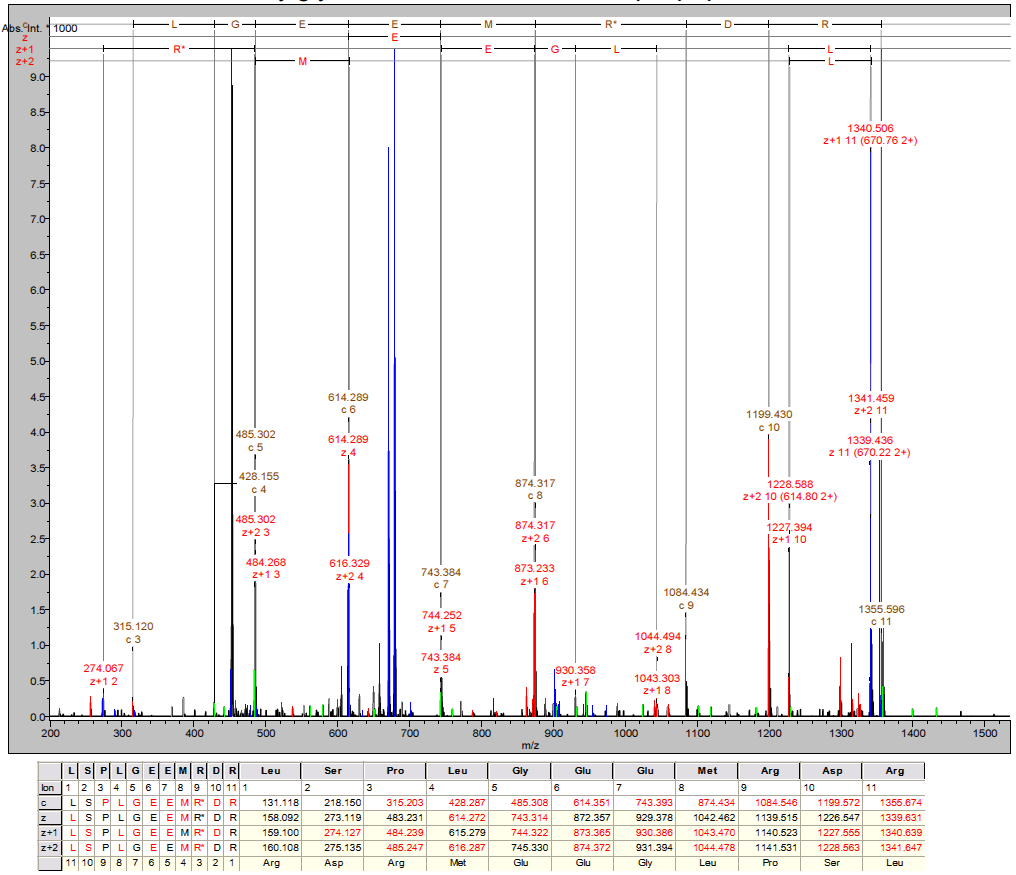
**

**Fig. S3.** Location of sites of hotspot glycation by methylglyoxal in apolipoprotein A1 by ETD ion trap mass spectrometry - glycation site at R149. Mass spectrum of z+1 and c ion series of amino acids 141 – 151. Methylglyoxal modification on R*. Biotools score 544.
